# The Role of EjSVPs in Flower Initiation in *Eriobotrya japonica*

**DOI:** 10.3390/ijms20235933

**Published:** 2019-11-26

**Authors:** Yuanyuan Jiang, Jiangrong Peng, Zhike Zhang, Shoukai Lin, Shunquan Lin, Xianghui Yang

**Affiliations:** 1 State Key Laboratory for Conservation and Utilization of Subtropical Agro-bioresources, College of Horticulture, South China Agricultural University, Wushan Road 483, Tianhe District, Guangzhou 510642, China; yyjiang613@163.com (Y.J.); jiangrongp@163.com (J.P.); poloky2@163.com (Z.Z.); 2 Key Laboratory of Loquat Germplasm Innovation and Utilization (Putian University), Fujian Province University, Putian 351100, China; shoukai.lin@foxmail.com

**Keywords:** SVP, loquat, flowering, dormancy, MADS-box

## Abstract

Flowering plants have evolved different flowering habits to sustain long-term reproduction. Most woody trees experience dormancy and then bloom in the warm spring, but loquat blooms in the cold autumn and winter. To explore its mechanism of flowering regulation, we cloned two *SHORT VEGETATIVE PHASE (SVP)* homologous genes from ‘Jiefanzhong’ loquat (*Eriobotrya japonica* Lindl.), namely, *EjSVP1* and *EjSVP2*. Sequence analysis revealed that the EjSVPs were typical MADS-box transcription factors and exhibited a close genetic relationship with other plant SVP/DORMANCY-ASSOCIATED MADS-BOX (DAM) proteins. The temporal and spatial expression patterns showed that *EjSVP1* and *EjSVP2* were mainly expressed in the shoot apical meristem (SAM) after the initiation of flowering; after reaching their highest level, they gradually decreased with the development of the flower until they could not be detected. *EjSVP1* expression levels were relatively high in young tissues, and *EjSVP2* expression levels were relatively high in young to mature transformed tissues. Interestingly, *EjSVP2* showed relatively high expression levels in various flower tissues. We analyzed the *EjSVP* promoter regions and found that they did not contain the C-repeat/dehydration-responsive element. Finally, we overexpressed the *EjSVPs* in wild-type *Arabidopsis thaliana* Col-0 and found no significant changes in the number of rosette leaves of *Arabidopsis thaliana*; however, overexpression of *EjSVP2* affected the formation of *Arabidopsis thaliana* flower organs. In conclusion, *EjSVPs* were found to play an active role in the development of loquat flowering. These findings may provide a reference for exploring the regulation mechanisms of loquat flowering and the dormancy mechanisms of other plants.

## 1. Introduction

The transformation of flowering plants from the vegetative stage to the reproductive stage results from coordinating the endogenous and exogenous environments. For successful reproduction, plants have evolved complex regulatory networks to ensure that plants bloom at the right time; these regulatory elements include the photoperiod, age, autonomic, vernalization, and gibberellin pathways [1,2,3,4,5]. These pathways are not independent but are linked to collectively regulate plant flowering transitions by a number of integrators such as FLOWERING LOCUS T (FT), SUPPRESSOR OF OVEREXPRESSION OF CONSTANS 1 (SOC1), and SHORT VEGETATIVE PHASE (SVP).

MIKC-type MADS-box transcription factors play an important role in the regulation of flower development and plant architecture. It contains the highly conserved MADS-box and K-box, and a relatively low degree of conserved I-domain and C-domain [6,7,8,9,10]. *SVP* is a member of the MIKC-type gene family and encodes a type II MADS-box protein containing the highly conserved MADS-box and K-box [8]. The *svp* mutant enables early flowering, and *SVP* is highly expressed during vegetative growth, delaying *Arabidopsis thaliana* flowering by inhibiting the expression of floral homeotic genes [11,12]. Studies in tomato plants have found that *svp* mutations inhibit tomato inflorescence growth [13].

In fruit trees, the MADS-box transcription factor *DORMANCY-ASSOCIATED MADS-BOX* (*DAM*) gene is involved in the regulation of dormancy. The *DAM* gene in fruit trees has the same sequence homology as *SVP* and is sometimes called the *SVP-like* gene [14]. In the study of Rosaceae fruit trees, such as apple [15,16,17,18,19,20,21], pear [22,23,24,25,26,27], peach [28,29,30,31,32], apricot [33,34,35], and sweet cherry [36,37], *SVP-like/DAM* genes were shown to maintain plant dormancy and have the highest expression levels during dormancy. *SVP-like/DAM* genes have also been identified in other plants, such as leafy spurge [38,39], kiwifruit [40], and aspen [41].

Loquat (*Eriobotrya japonica* Lindl.) belongs to the Rosaceae family and is an evergreen fruit tree planted in subtropical regions. Rosaceae contains the most abundant fruit crop species, such as apple, pear, peach, strawberry and loquat. In the Rosaceae family, the initiation and flowering of flower buds usually occur in different years, and flower buds need to undergo dormancy, such as for apples and pears [42]. Different from its close Rosaceae relatives such as apple and pear, the cultivated loquat (*Eriobotrya japonica*) blossoms from late fall into winter, and fruits ripen from late spring to summer. Its flower buds are continuously differentiated and do not undergo dormancy until flowering [43]. However, there are few reports on the regulation of loquat flowering. To date, *EjTFL1* [44], *EjLFY* [45], *EjAP1* [46], *EjFT* [47], and *EjSOC1* [43] have been identified from cultivated loquat; and *EdGI*, *EdCO*, *EdFT*, and *EdFD* have been cloned from wild loquat *Eriobotrya deflexa* Nakai forma *koshunensis* [48,49]. However, the cause of this unique flowering phenomenon remains unclear. More importantly, the function of the *SVP/DAM* genes in loquat has not been reported.

In this study, two *SVP* homologues were isolated from loquat, namely, *EjSVP1* and *EjSVP2*. To explore their role in the growth and development of loquat, we examined their expression patterns in different periods and different tissues of loquat and examined the effects of GA_3_ and short-day treatment on the expression of *EjSVPs*. Finally, we overexpressed *EjSVPs* in wild-type *Arabidopsis thaliana* Col-0 for functional analysis.

## 2. Results

### 2.1. Cloning and Identification of Loquat EjSVPs

Two *SVP* homologous genes were isolated from loquat: *EjSVP1* and *EjSVP2*. Their full-length coding sequences are 675 bp and they encode 224 amino acids (Supplementary Sequence S1 and S2). Their sequences are highly similar, with a nucleotide sequence identity of 92.74% and a predicted amino acid sequence identity of 86.61%.

**Figure 1:**
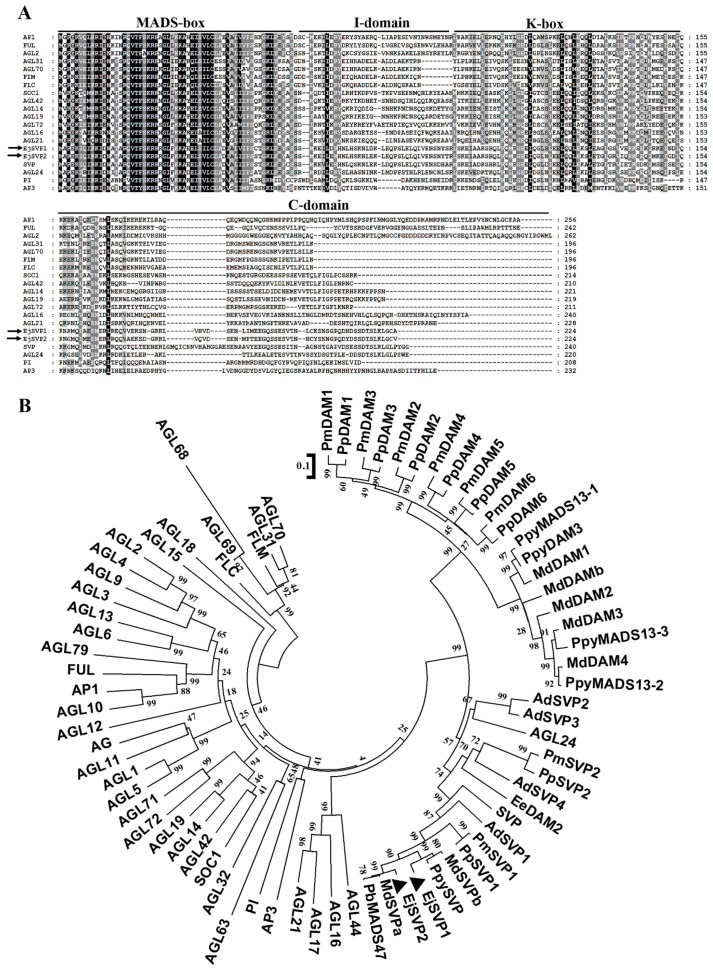
Amino acid sequence alignment and phylogenetic analysis of EjSVP proteins. (**A**) Alignment of the amino acid sequences of the EjSVP proteins with the members of the *Arabidopsis thaliana* MIKC-type transcription factor family. The black line marks the highly conserved MADS-box and K-box domains. The I-domain is behind the MADS-box, and the C-domain is behind the K-box. (**B**) Phylogenetic analysis of MIKC-type transcription factors from *Arabidopsis thaliana*, SVP-like/DAM, and EjSVPs. The protein sequences were retrieved from NCBI (for accession IDs, see the Supplementary ID information).

As described above, the MIKC-type MADS-box transcription factor includes four structures: the MADS-box, I-domain, K-box and C-domain. Sequence analysis showed that both EjSVP proteins have four typical structures: the MADS-box, I-domain, K-box and C-domain (Figure 1A).

According to the results of the phylogenetic tree, EjSVP1 and EjSVP2 are clustered with the SVP-like/DAM proteins of other plants. More importantly, EjSVPs and the Maloideae SVP-like/DAM protein clustered in a small clade (Figure 1B).

### 2.2. Tissue-Specific Expression Patterns of EjSVPs in Loquat

**Figure 2:**
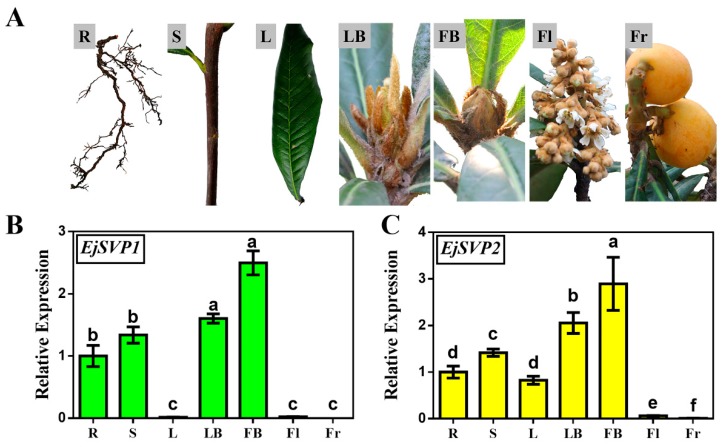
Tissue-specific expression levels of *EjSVP1* and *EjSVP2.* (**A**) Different tissues from loquat. (**B**,**C**) Relative expression levels of *EjSVP1* and *EjSVP2* in the different tissues shown in (**A**) were measured by qRT-PCR (error bars indicate Standard Error(SE) from three biological replicates). The *Ejβ-actin* gene served as an internal control. The relative expression levels were expressed as the ratio of the expression levels between the *EjSVP* genes and the reference gene, so as to indicate the relative transcript levels of the genes compared the levels of *Ejβ-actin*. An expression value of 1 was assigned to the first sample. R, root; S, shoot; L, leaf (May 26); LB, leaf bud (May 26); FB, flower bud (August 18); Fl, flower (December 8); Fr, fruit (March 30). Different letters on bar indicate significant difference (*p* < 0.05) by student’s *t*-test.

To explore the potential functions of *EjSVP1* and *EjSVP2* in loquat, the transcription levels of *EjSVP1* and *EjSVP2* in various tissues of ‘Jiefangzhong’ were investigated, including the roots, stems, leaves, leaf buds, flower buds, flowers, and fruit (Figure 2A).

qRT-PCR results showed that *EjSVP1* and *EjSVP2* were mainly expressed in the roots, stems, leaf buds and flower buds (Figure 2B, C). Both genes were slightly expressed in the inflorescences. *EjSVP1* was hardly expressed in mature leaves (Figure 2B), while the expression level of *EjSVP2* was relatively high (Figure 2C). The expression patterns of *EjSVP1* and *EjSVP2* in mature leaves were significantly different, indicating that the two genes may perform different functions during the growth and development of loquat.

### 2.3. Temporal and Spatial Expression Patterns of EjSVPs in Loquat

To further clarify the roles of *EjSVP1* and *EjSVP2* in loquat, we analyzed the expression levels of *EjSVPs* in the shoot apical meristems (SAM) (different developmental stages), fruit (different developmental stages), flower (different tissues) and leaves (different maturities in the same period; see Supplementary Figure S1).

**Figure 3:**
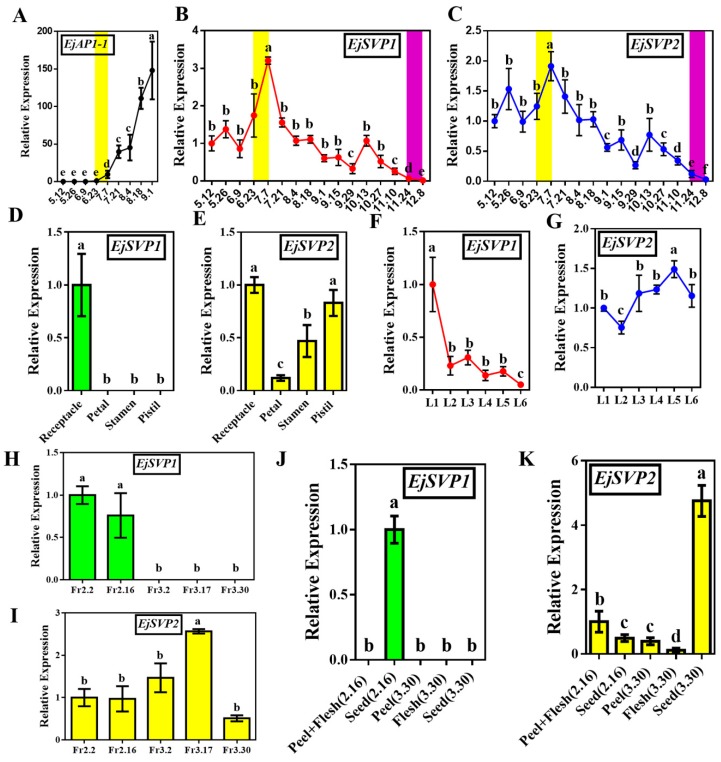
Expression patterns of *EjSVPs* during loquat growth and development. (**A**) Expression trend of *EjAP1-1* in the SAM from May to September. (**B**,**C**) Expression trend of *EjSVP1* and EjSVP2 in the SAM from May to December. (**D**,**E**) Relative expression levels of *EjSVP1* and *EjSVP2* in different flower tissues. (**F**,**G**) Relative expression levels of *EjSVP1* and *EjSVP2* in leaves at different levels of maturity in the same period. (**H**,**I**) Expression trend of *EjSVP1* and *EjSVP2* during fruit development. (**J**,**K**) Relative expression levels of *EjSVP1* and *EjSVP2* in different fruit tissues at different developmental stages. The *Ejβ-actin* gene served as an internal control. The relative expression levels were expressed as the ratio of the expression levels between the *EjSVP* genes and the reference gene, so as to indicate the relative transcript levels of the genes compared the levels of *Ejβ-actin*. An expression value of 1 was assigned to the first sample. The yellow background (in A, B, and C) indicates the initiation period of flower budding and the purple background indicates flower opening. Error bars indicates ± SE from three biological replicates. Different letters on bar indicate significant difference (*p* < 0.05) by student’s *t*-test.

The ortholog gene *EjAP1-1* of the floral meristem identity gene *AP1* has been confirmed as a marker gene for identifying loquat floral bud differentiation [43]. In this experiment, we found a high level of *EjAP1-1* expression after 23 June (Figure 3A), which is consistent with the results of Jiang et al. [43]. This finding indicates that the flower bud differentiation of loquat occurs towards the end of June and in early July.

The expression trends of *EjSVP1* and *EjSVP2* were similar during flower development in SAM. The expression levels of the two genes were relatively high after the initiation of flower bud differentiation. With the development of flower buds, their expression levels gradually decreased until they were almost undetectable in open flowers (Figure 3B,C). In different tissues of the flowers (not opened), *EjSVP1* was expressed only in the receptacle, while *EjSVP2* was expressed in various tissues (Figure 3D,E).

*EjSVP1* was mainly expressed in L1–L3 (the leaves at different maturities in the same period) and was almost undetectable after leaf maturity (L6) (Figure 3F), while *EjSVP2* was expressed in several stages of leaf development (Figure 3G). Moreover, as the leaves developed, the expression level of *EjSVP2* gradually increased and then slightly decreased after maturity (Figure 3G). Similarly, during fruit development, *EjSVP1* was mainly expressed in the young fruit stage (Fr2.2–Fr2.16) but only in the seeds (Figure 3H,J), while *EjSVP2* was relatively highly expressed during the whole developmental period, and the peak expression occurred during the fruit expansion period (Fr3.2–Fr3.17) (Figure 3I). Regarding different fruit tissues and different periods, *EjSVP2* was mainly expressed in the flesh and peel of young fruit (the peel could not be separated at this stage), and expressed in the seeds of mature fruits (Figure 3K). Previous studies have shown that the leaves undergo intense cell division during the L1–L2 period [50,51].

### 2.4. Effects of Exogenous GA_3_ Treatment and Short-Day Treatment on the Expression of EjSVPs

**Figure 4:**
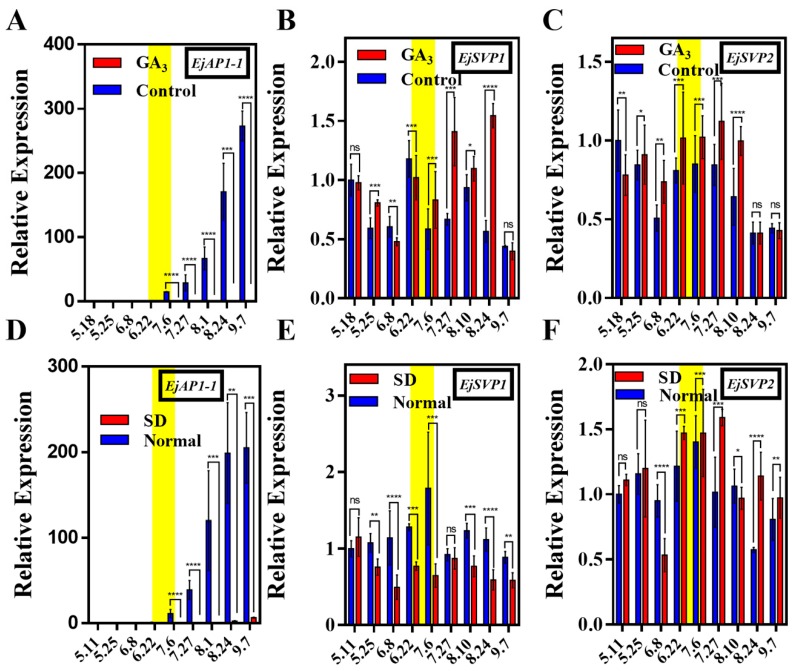
Effect of GA_3_ treatment and SD treatment on *EjSVP1* and *EjSVP2* expression in loquat SAM. (**A**) The expression trends of *EjAP1-1* in the GA_3_ treatment and control groups. (**B**) and (**C**) The expression trends of *EjSVP1* and *EjSVP2* in the gibberellin treatment and control groups. (**D**) The expression trends of *EjAP1-1* in the short-day treatment and control groups. (**E**) and (**F**) The expression trends of *EjSVP1* and *EjSVP2* in the short-day treatment and control groups. The *Ejβ-actin* gene served as an internal control. The relative expression levels were expressed as the ratio of the expression levels between the *EjSVP* genes and the reference gene, so as to indicate the relative transcript levels of the genes compared the levels of *Ejβ-actin*. An expression value of 1 was assigned to the first sample. The experimental treatment time was from 18 May to 10 August. The yellow background (in A, B, and C) indicates the initiation period of flower budding. Error bars indicates ± SE from three biological replicates. ‘ns’ indicates that the difference is not significant between treatment groups and control (or normal) groups; asterisks (*) indicate significant between treatment groups and control (or normal) groups, **** *p <* 0.0001, *** *p* < 0.001, ** *p* < 0.01, * *p* < 0.05, by student’s *t*-test.

Loquat could not undergo flower bud differentiation after GA_3_ and short-day (SD) treatments. To explore the effects of two treatments on *EjSVPs*, the transcription levels of *EjAP1-1*, *EjSVP1* and *EjSVP2* were detected. *EjAP1-1* was strongly inhibited after GA_3_ and SD treatment (Figure 4A,D), and this result is consistent with Jiang et al. [43]. Compared with the control group, the expression levels of *EjSVP1* and *EjSVP2* were higher in the days after GA_3_ treatment but returned to the control group when stopping treatment (Figure 4B,C). The expression levels of *EjSVP1* were decreased after SD treatment, while *EjSVP2* expression levels did not show significant changes (Figure 4E,F).

### 2.5. Subcellular Localization of EjSVPs

**Figure 5:**
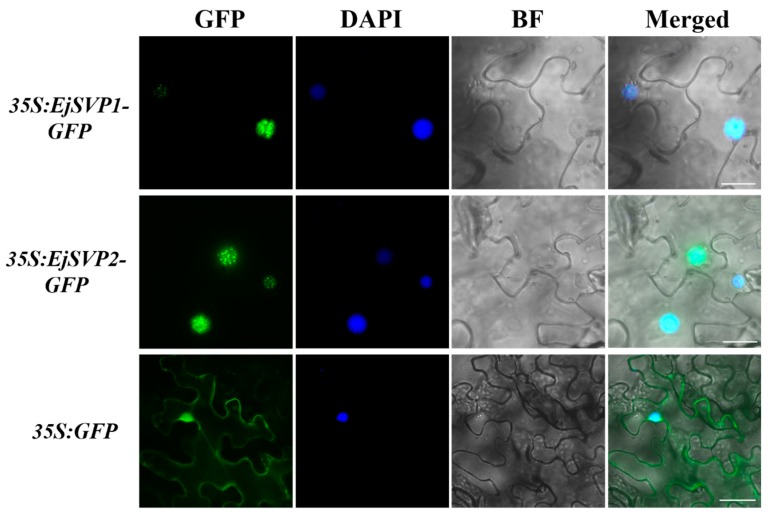
Subcellular localization of EjSVPs. GFP, GFP fluorescence; 4,6-diamidino-2-phenylindole (DAPI) staining indicates nuclear localization; bright-field (BF); merged image of GFP, BF and DAPI (Merged). Scale bars = 50 µm. 35S: GFP as a control.

To determine the cellular localization of the encoded EjSVP proteins, two fusion expression vectors, 35S:EjSVP1-GFP and 35S:EjSVP2-GFP, were constructed and transferred into the epidermis of tobacco plant cells to observe the fluorescence signal. The green fluorescence of the 35S:EjSVP1-GFP and 35S:EjSVP2-GFP fusion proteins were detected in the nucleus (Figure 5), indicating that EjSVP1 and EjSVP2 are localized in the nucleus. This result is consistent with the characteristics of general transcription factors.

### 2.6. Promoter Analysis of EjSVPs

Previous studies have shown that the C-repeat/dehydration-responsive element (CRT/DRE) is a cold response element found in leafy spurge [39], Japanese pear [27,52], apple [16,17] and Japanese apricot [35]. C-repeat binding factors (CBFs) increase rapidly at low temperatures [16,17,27,34,35,53,54]. CBF recognizes and binds to the CRT/DRE motif to regulate the expression of *SVP-like*/*DAM* genes.

To explore why *EjSVPs* possess this expression pattern that is different from other dormant plants, their promoter (sequence information was obtained from the de novo genome sequencing project of loquat) structural elements were analyzed. No CCGAC structural elements (CRT/DRE) were found in the promoter regions of the two *EjSVPs* (Supplementary Sequence S3, S4). Interestingly, one CCAAT structural element was found in the promoter region of *EjSVP1* (Supplementary Sequence S3), and four CCAAT structural elements were found in the promoter region of *EjSVP2* (Supplementary Sequence S4). The cis-element CCAAT DNA-binding motif is a structural element that responds to the key gene *CO* of the photoperiod pathway and is called *CORE* (CO response element) [55,56]. Interestingly, *EjSVPs* began to be highly expressed after 23 June (Figure 3B,C), which is the longest day of sunshine in Guangzhou.

In addition, AuxRR-core (GGTCCAT) was also found in the promoter region of *EjSVP1* but was not found in the promoter region of *EjSVP2* (Supplementary Sequence S3), which may be the reason for the relatively high expression of *EjSVP1* in tender tissues.

### 2.7. Functional Analysis of EjSVPs in Arabidopsis thaliana

**Figure 6:**
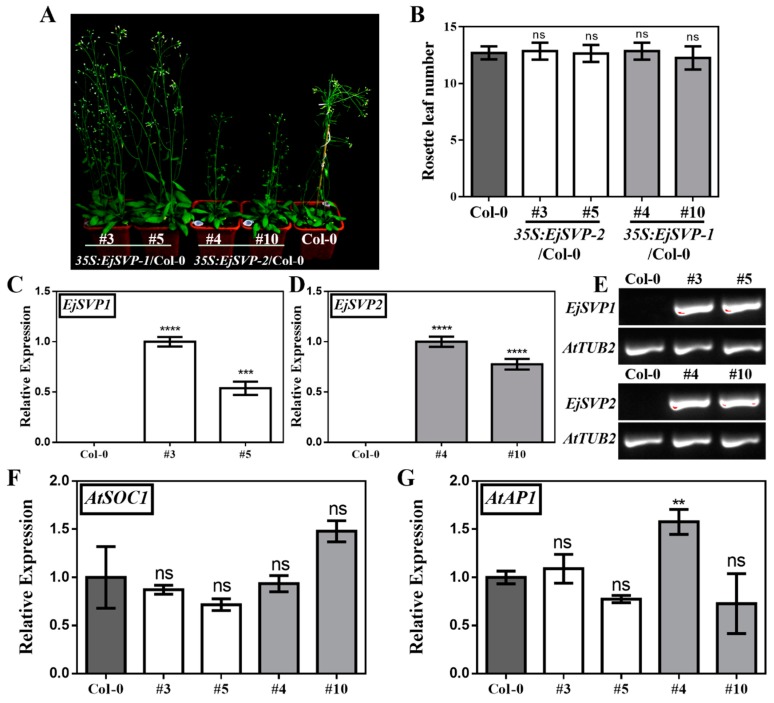
Effect of *EjSVP* overexpression on *Arabidopsis thaliana* flowering. (**A**) Forty-day-old 35S:*EjSVP* transgenic lines and Col-0 phenotype. (**B**) The number of rosette leaves of Col-0 and *35S:EjSVP* transgenic plants. *n* = 20; the error bars represent the SD from *n* = 20, by student’s *t*-test. (**C**) and (**D**) *EjSVP* expression in twenty-day-old Col-0 and *35S:EjSVP* transgenic plants, error bars indicate ± SE from three biological replicates. (**E**) RT-PCR results of *EjSVP* in twenty-day-old transgenic plants. (**F**,**G**) Expression of *AtSOC1* and *AtAP1* in twenty-day-old Col-0 and *35S:EjSVP* transgenic plants. *AtTUB2 (AT5G62690)* served as an internal control. The relative expression levels were expressed as the ratio of the expression levels between the genes and the reference gene, so as to indicate the relative transcript levels of the genes compared the levels of *AtTUB2*. An expression value of 1 was assigned to the first sample. Error bars indicate ± SE from three biological replicates. ‘ns’ indicates that the difference is not significant between transgenic lines and Col-0; asterisks (*) indicate significant between transgenic lines and Col-0, **** *p <* 0.0001, *** *p* < 0.001, ** *p* < 0.01, by student’s *t*-test.

We overexpressed the *EjSVP* genes in wild-type *Arabidopsis thaliana* Col-0 and found that the number of rosettes of T3 generation *35S:EjSVP1* and *35S:EjSVP2* transgenic lines were similar to that of wild-type Col-0 (Figure 6A,B). We then performed qRT-PCR and RT-PCR analysis of *EjSVP* in the transgenic lines and found that *EjSVP* were abundantly expressed in the transgenic lines (Figure 6C–E). In addition, we performed qRT-PCR analysis of *AtSOC1* and the floral meristem identity gene *AtAP1* in *Arabidopsis thaliana*, and the results showed that the *AtSOC1* expression levels in the transgenic lines were not significantly different from that in Col-0 (Figure 6F). The expression level of *AtAP1* was not significantly different between the *35S:EjSVP1* transgenic lines and the Col-0 line. The 35S:EjSVP2 transgenic line #4 exhibited significant higher expression of *AtAP1* compared to wild type, while no difference could be observed in line #10 (Figure 6A,B,G).

**Figure 7:**
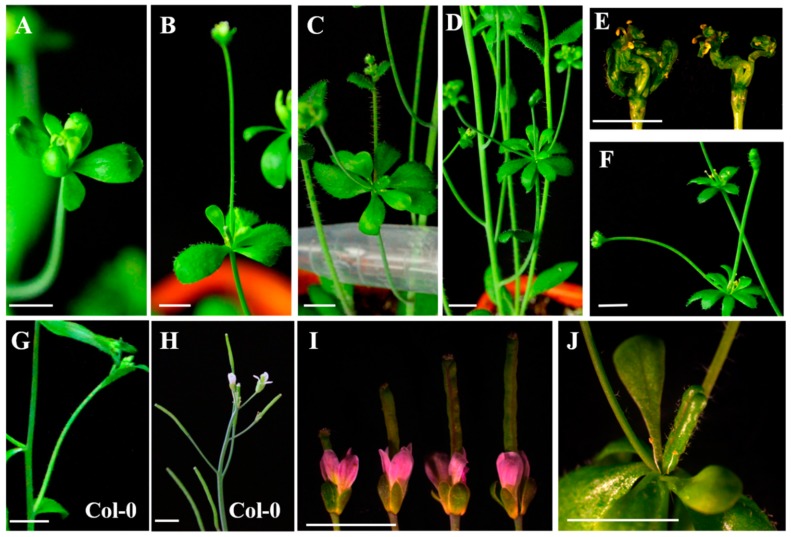
Phenotypes of flowers and siliques of Col-0 and *35S:EjSVP* transgenic plants. (**A**–**D**,**F**,**I**,**J**) Variations in the flowering of *EjSVP2*-overexpressing transgenic lines. (**E**) Cross-sectional view of the secondary flowers in (**D**,**F**). (**G**,**H**) Flowering traits of wild-type *Arabidopsis thaliana* Col-0. Scale bars = 5 mm.

Interestingly, compared to the wild type *Arabidopsis thaliana* Col-0 phenotype (Figure 7G,H), in the *35S:EjSVP2* transgenic line, various types of floral organ variations were produced, including flowers that had bract-like sepals (Figure 7A–D,F,J), stunted stamens (Figure 7B–E,J), the formation of a secondary flower (Figure 7B–F), and petals that accumulated pigment (Figure 7I). However, the *35S:EjSVP1* transgenic line did not have any of these features.

## 3. Discussion

Rosaceae woody fruit trees are extremely important in peoples’ lives and bring great benefits to growers every year. Loquat belongs to the Maloideae family, and its flowering habit is unique for woody fruit trees. It is interesting and meaningful to explore the reasons for its different flowering habits compared to apples and pears. Moreover, the flowering times among the 26 species of *Eriobotrya* are different [57]. For example, *Eriobotrya deflexa* Nakai blooms in spring, similar to apples [48,49,58]. The flowering habits and varying germplasms in *Eriobotrya* have demonstrated that loquat could be an ideal model material for the study of Rosaceae flowering shifts to adapt to climate changes. Studying the regulation mechanism of loquat flowering could provide a favorable reference for the flowering regulation of other woody plants.

Our results show that EjSVPs and MdSVPs form a small clade with a high genetic relationship (Figure 1B), suggesting that they are more similar in structure and closer in genetic relationship, and thus suggest that EjSVPs could have similar functions to the SVP-like/DAM proteins of Maloideae. However, the *SVP/DAM* gene in apples is mainly involved in dormancy [15,16,17,18,19,20,21]. Jiang et al. [43] showed that loquat did not undergo dormancy, but rather its flower bud differentiation was continuous and blossomed in autumn and winter. The expression pattern of *EjSVPs* is also different from the orthologous genes in apples [21]. Additionally, analysis of promoter cis-structural elements found that the promotor of *EjSVPs* is different from other plants and does not contain *CRT/DRE* (Supplementary Sequence S3, S4). These findings indicate that loquat produces a unique pattern of flowering regulation during natural selection. However, the specific reasons for these flowering characteristics, as well as the functional changes of *EjSVPs*, require further progress.

*EjSVP1* expression levels were relatively high in young tissues, and *EjSVP2* expression levels were relatively high in young to mature transformed tissues (Figure 3). Furthermore, exogenous GA_3_ could strongly inhibit the expression of *EjSOC1s* and *EjAP1s*, and the SAM showed strong vegetative growth, while the stem tip of the short-day treatment group almost stopped growing, and the loquat could not undergo flower bud differentiation [43]. Our results show that GA_3_ treatment promotes the expression of *EjSVPs*, whereas SD treatment has some inhibitory effects on the expression of *EjSVP1* (Figure 4). Based on these results, we speculated that EjSVP1 may regulate cell proliferation, and EjSVP2 may be involved in cell expansion.

In *Arabidopsis thaliana*, *SVP* genes are expressed at relatively high levels in the roots and leaves during vegetative growth, while they are expressed in low amounts in inflorescences, and hardly expressed in flowers and siliques [11]. However, in this experiment, *EjSVPs* were found to be highly expressed in flower buds (Figure 2B,C). In the *35S:EjSVP2* transgenic line, the flowers and siliques produced variations, such as flowers that had bract-like sepals, stunted stamens, the formation of secondary flowers, and petals that accumulated pigment (Figure 7). However, these phenotypes were not found in the *35S:EjSVP1* transgenic line. Combined with the qRT-PCR results from loquat flowers, *EjSVP1* was found to be only expressed in receptacles, while *EjSVP2* was expressed in receptacles, petals, and stamens, especially in the pistils. These findings suggest that *EjSVP2* actively participates in the morphogenesis of flowers in loquats.

In this study, the *35S:EjSVP2* transgenic line showed a phenotype similar to the 35S:AGL24 transgenic line (Figure 7A–F) [59,60]. In addition, phylogenetic tree analysis found that EjSVPs and AtAGL24 were clustered together (Figure 1B). Interestingly, in *Arabidopsis thaliana*, the heterodimerization of SOC1 and AGL24 activates the expression of the downstream floral meristem identity gene *LFY* and promotes flowering [60,61]. More importantly, in a study of Japanese apricot, PmDAM6 (SVP-like) was found to interact with PmSOC1 [62]. Moreover, EjSOC1 in loquat has also been verified to play an active role in flower regulation, and the expression pattern in SAM was similar to that of EjSVPs (Figure 2B,C) [43]. We hypothesize that in loquat, EjSVP2 may be more similar to the regulation pattern of AGL24 in *Arabidopsis thaliana*, where it interacts with EjSOC1 to regulate the development of loquat flowering.

The *SVP* gene also plays an important role in response to temperature signals, which inhibits flowering by negatively regulating *FT* expression [63]. SVP can also promote *EARLY FLOWERING MYB PROTEIN (EFM)* expression, and EFM can regulate flowering in response to temperature-suppressed FT expression [64]. In addition, when the temperature is changed from 16 °C to 27 °C, more FLOWERING LOCUS M (FLM)-δ-SVP complexes are formed, which hinders FLM-β and SVP from inhibiting flowering. Higher temperatures can degrade SVP protein, and then SVP-FLM-β complexes are reduced, thereby activating downstream target genes such as *FT* and *SOC1* expression, promoting flowering [65]. Interestingly, from May to July, the temperature rose (Supplementary Figure S2A), and the expression of *EjSVPs* began to rise sharply and peak (Figure 3B,C). On the other hand, the phytohormone abscisic acid (ABA) plays a key role in response to stress such as drought. Under long-days and drought conditions, GIGANTEA (GI) can positively regulate the expression of *FT* and *SOC1* to promote flowering, and requires the participation of ABA, independent of CO; in short-days, SVP can prevent ABA from activating *SOC1* [66]. Importantly, there was more rain in May–June, and the rain was greatly reduced in July (Supplementary Figure S2B). These results suggested that *EjSVPs* may be involved in the regulation of loquat flowering by temperature signal or drought stress.

## 4. Materials and Methods

### 4.1. Plant Materials and Growth Conditions

Twelve-year-old ‘Jiefanzhong’ loquat (*Eriobotrya japonica* Lindl.) were used in each experiment. The trees were located at the loquat germplasm resource preservation garden (South China Agricultural University, Guangzhou, China), and showed normal flowering. Selected disease-free plant tissues (roots, stems, leaves, buds, flowers and fruits) with consistent phenotypes were collected at each sampling (tissues were taken at 4 p.m., every two weeks). Samples for qRT-PCR analysis were frozen in liquid nitrogen and then stored in an ultralow temperature freezer at −80 °C until use. *Arabidopsis thaliana* wild-type Col-0 and *Nicotiana benthamiana* were used in this study and grown under long-day conditions (16 h light/8 h dark cycle) at 22 °C.

### 4.2. RNA Extraction, Reverse-Transcription, Gene Isolation, and Sequence Analysis

Total RNA was extracted using an EASY Spin Plus Plant RNA Extraction kit (Aidlab, Beijing, China). First-strand cDNA was generated from loquat bud RNA using a PrimeScript^TM^ RT reagent kit with gDNA Eraser (TaKaRa, shiga, Japan), following the manufacturer’s instructions.

The full-length coding sequences of *EjSVP1* and *EjSVP2* were isolated from first-strand cDNA using Prime STAR^®^ Max DNA Polymerase (TaKaRa, shiga, Japan). The two primers for *EjSVP1* were 5′-ATGGCGAGGGAGAAGATTCAGAT-3′ and 5′-TTAAGCGCACCCCAATTTTAGAGA-3′; and the two for *EjSVP2* were 5′-ATGGCGAGGGAGAAAATTCAGAT-3′ and 5′-TTAAACGCACCCCAATTTTAGAGA-3′. The PCR conditions were carried out in strict accordance with the reagent instructions. Sequence information was obtained from the de novo genome sequencing project of loquat, which has not yet been published. Sequence analysis was carried out according to a previous method [43]. Predictive analysis of cis-acting elements of the promoter of *EjSVPs* was performed online at and PLACE (https://www.dna.affrc.go.jp/PLACE/?action=newplace) and PlantCARE (http://bioinformatics.psb.ugent.be/webtools/plantcare/html/).

### 4.3. Gene Expression Analysis

Quantitative real time polymerase chain reaction (qRT-PCR) using iTaqTM universal SYBR Green Super mix kit (Bio-Rad, Hercules, CA, USA) was carried out in triplicate using a LightCycler^R^ 480 system (Roche, Basel, Switzerland). The relative expression levels were all evaluated by the 2^−ΔΔCt^ (cycle threshold) method [67]. *Ejβ-Actin* was used as an internal control for loquat [68], and *AtTUB2 (AT5G62690)* for *Arabidopsis thaliana* [64]. The relative expression levels were expressed as the ratio of the expression levels between the genes and the reference gene, so as to indicate the relative transcript levels of the genes compared the levels of *Ejβ-actin* or *AtTUB2*. The primer sequences used in qRT-PCR for *EjAP1-1* have been described by Jiang et al. [43]. Two primers were used for *EjSVP1*, 5′-GAAGTCCCTTGAAGCTGGCT-3′ and 5′-CTCTTCCGCCAATTGCATCG-3′; and two for *EjSVP2*, 5′-TCCCTTGAAACTGGCTTGGG-3′ and 5′-CTCCGCCACTTGCTGTCTTA-3′. The primer sequences used in RT-PCR for *EjSVPs* were the same as the cloning primers (stop codon removed). The PCR conditions were carried out in strict accordance with the reagent instructions. The RT-PCR product was detected by agarose gel electrophoresis (10 mg mL^−1^). Data were collected from three biological replicates.

### 4.4. Short-Day and GA_3_ Treatments

For short-day (SD) treatment, the natural light was set for 8 h (10 a.m.–6 p.m.), with full darkness for 16 h (6 p.m.–10 a.m. the following day) using shading. The control plants were grown naturally. In the GA_3_ treatment, 300 mg L^−1^ of GA_3_ aqueous solution (Dingguo, Beijing, China) was sprayed onto the plants every two weeks, and 0.1% (v/v) phosphoric acid and 0.025% (v/v) Triton X-100 were added as surfactants. The control plants were sprayed with an aqueous solution containing the surfactants. The experimental treatment time was from 18 May 2018 to 10 August 2018. The SAM of the treatment groups and the control groups was used for qRT-PCR analysis, and the sampling method was the same as in Section 4.1.

### 4.5. Subcellular Localization and Arabidopsis thaliana Transformation

*EjSVP1* and *EjSVP2* were cloned into pGreen-35S-GFP [69] for subcellular localization analysis and into pGreen-35S [69] for *Arabidopsis thaliana* transformation. Each construct was introduced into *Agrobacterium tumefaciens* GV3101::pSoup and then transformed into *Arabidopsis thaliana* Col-0 using the floral dip method [70] or the transient transformation of *N. benthamiana* leaves [71]. Transgenic seedlings were selected on soil using Basta (3.5 mg L^-1^). Fluorescence microscopy (Observer D1, Zeiss, Jena, Germany) was used to detect the fluorescence signal of the EjSVP-GFP fusion proteins.

### 4.6. Statistics and Analysis

Microsoft Office Excel was used to analyze the data, and the difference between the data was assessed by student’s *t* test. Graph production was performed using Microsoft Office PowerPoint and GraphPad Prism 6 software.

## 5. Conclusions

In this study, we identified and named two *EjSVP* genes from loquat. Through expression analysis during flower development, we found that two *EjSVP* genes play an active role in flower development. At the same time, temporal and spatial expression analysis and GA_3_ and SD treatment results suggest that *EjSVPs* may be involved in cell division or growth. In addition, overexpression of *EjSVPs* to wild type *Arabidopsis thaliana* Col-0 had no significant effect on the change of rosette leaves. The promoter region of *EjSVP* genes in loquat does not contain CRT/DRE, which may be the main reason that the flowering habits of loquat differ from other fruit trees in the Rosaceae family, which also provides an important clue for the study of loquat flower regulation.

## Supplementary Materials

Supplementary materials can be found at https://www.mdpi.com/1422-0067/20/23/5933/s1.
