# Supplementary material for: The Role of EjSVPs in Flower Initiation in Eriobotrya japonica
Source: Int J Mol Sci. 2019 Nov 26;20(23):5933. doi: 10.3390/ijms20235933 (PMC6928820; doi:10.3390/ijms20235933)
Supplement: Supplementary file 1 [file ijms-20-05933-s001.zip › revision-2-Supplementary Materials.docx]

The role of EjSVPs in flower initiation in *Eriobotrya japonica*

Yuanyuan Jiang^1,2,3#^, Jiangrong Peng^3#^, Zhike Zhang^3^, Shoukai Lin^2^, Shunquan Lin^3*^, and Xianghui Yang^3*^

**Supplementary ID information.** Protein name and accession IDs from NCBI:

AG (AT4G18960), AGL1 (AT3G58780), AGL2 (AT5G15800), AGL3 (AT2G03710), AGL4 (AT3G02310), AGL5 (AT2G42830), AGL6 (AT2G45650), AGL9 (AT1G24260), AGL10 (AT1G26310), AGL11 (AT4G09960), AGL12 (AT1G71692), AGL13 (AT3G61120), AGL14 (AT4G11880), AGL15 (AT5G13790), AGL16 (AT3G57230), AGL17 (AT2G22630), AGL18 (AT3G57390), AGL19 (AT4G22950), AGL21 (AT4G37940), AGL24 (AT4G24540), AGL31 (AT5G65050), AGL32 (AT5G23260), AGL42 (AT5G62165), AGL44 (AT2G14210), AGL63 (AT1G31140), AGL68 (AT5G65080) , AGL69 (AT5G65070), AGL70 (AT5G65060), AGL71 (AT5G51870), AGL72 (AT5G51860), AGL79 (AT3G30260)., AP1 (AT1G69120), AP3 (AT3G54340), FLC (AT5G10140 ), FLM (AT1G77080), FUL (AT5G60910), PI (AT5G20240), SOC1 (AT2G45660), SVP (AT2G22540), AdSVP1 (AFA37963), AdSVP2 (AFA37964), AdSVP3 (AFA37965), AdSVP4 (AFA37966), EeDAM2 (ABY53594), MdDAM1 (AOA32865), MdDAM2 (AOA32866), MdDAM3 (XP_017186028), MdDAM4 (AOA32868), MdDAMb (ADL36743), MdSVPa (AOA32867), MdSVPb (BAR40332) , PbMADS47 (XP_009364259), PmDAM1 (BAK78921), PmDAM2 (BAK78922), PmDAM3 (BAK78923), PmDAM4 (BAK78924), PmDAM5 (BAK78920), PmDAM6 (BAH22477), PmSVP1 (AML81015), PmSVP2 (AML81016), PpDAM1 (ABJ96361), PpDAM2 (ABJ96363), PpDAM3 (ABJ96364), PpDAM4 (ABJ96358), PpDAM5 (ABJ96359), PpDAM6 (ABJ96360), PpSVP1 (XP_020422316), PpSVP2 (XP_020409383), PpyMADS13-1 (BAI48074), PpyMADS13-2 (BAI48075), PpyMADS13-3 (BAM74166), PpyDAM3 (AJW29049), PpySVP (AJW29050).


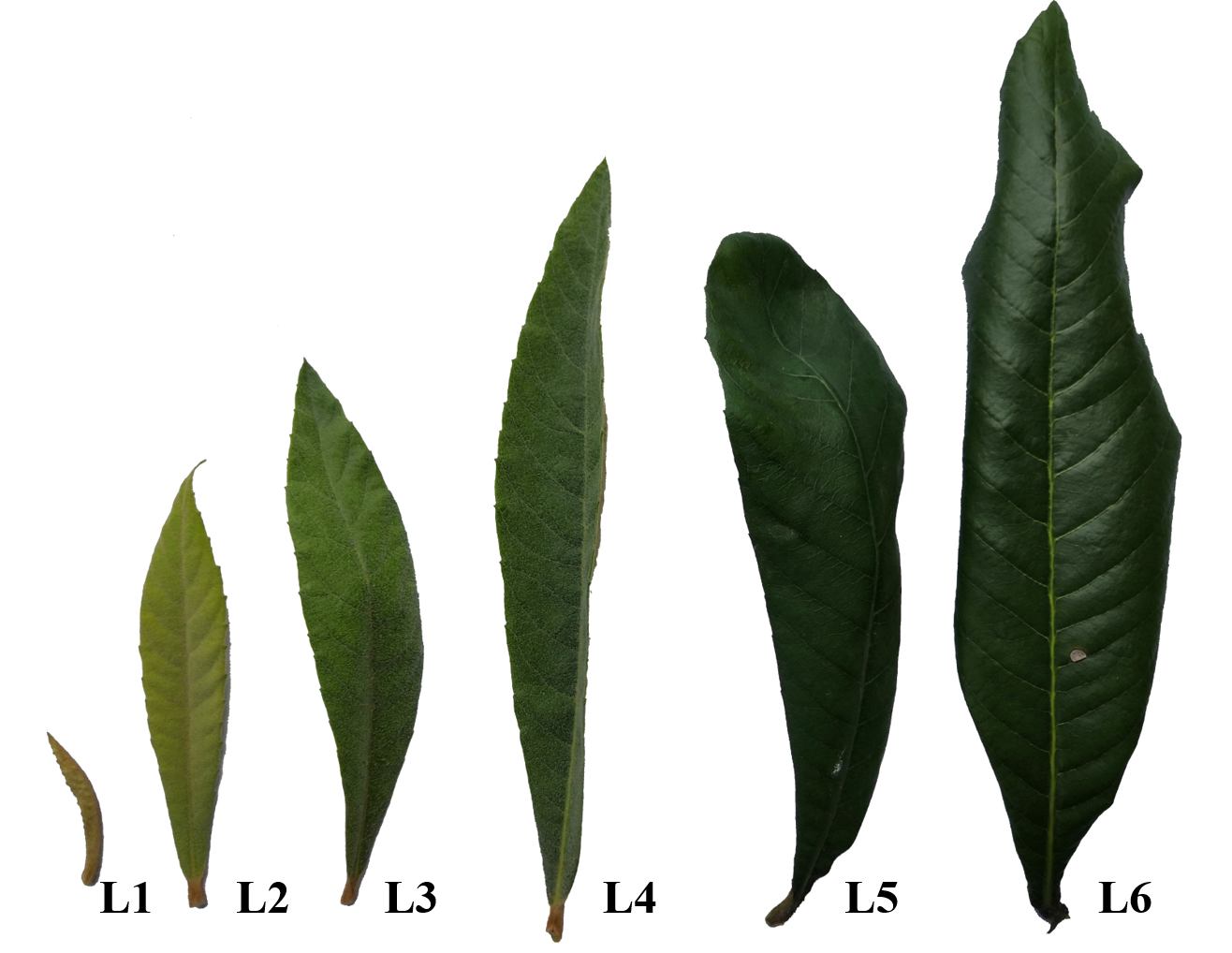


**Figure S1. Leaves of** **different** **maturities in the same period.** L1, L2, L3, L4, L5 and L6 were taken from leaves of different maturities on June 9th, ‘L6’ indicated in maturity stage of leaves.


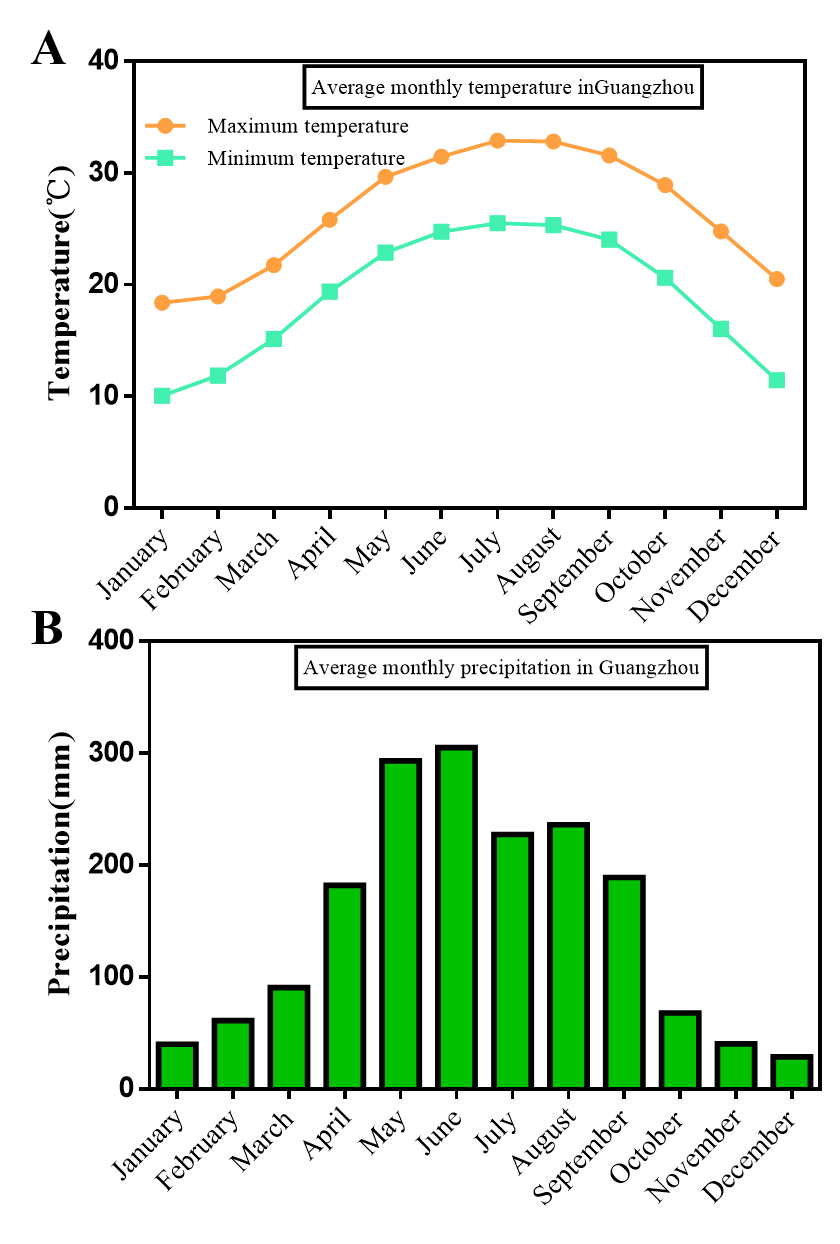


**Figure S2. Average monthly temperature and precipitation in Guangzhou.** (A) Average monthly temperature. (B) Average monthly precipitation. Data from China Central Meteorological Observatory (http://www.nmc.cn/publish/forecast/AGD/guangzhou.html).

**Supplementary** **sequence**

**Sequence S1.** Amino acid sequence information of *EjSVP1* gene and encoded amino acid sequence

**1 ATGGCGAGGGAGAAGATTCAGATCAAGAAGATCGACAACGCGACAGCGAGGCAGGTGACCTTTTCCAAGAGGAGAAGAGGCCTTTTCAAGAAGGCT**

**1 M A R E K I Q I K K I D N A T A R Q V T F S K R R R G L F K K A**

**97 GAGGAGCTCTCCATTCTCTGTGATGCCGATATTGCTCTTGTCATCTTTTCTTGCACCGGAAAGCTCTTTGAATACTCCAGCTCTAGTATGAAGGAA**

**33 E E L S I L C D A D I A L V I F S C T G K L F E Y S S S S M K E**

**193 ATTCTAGACAGGCACAACTTGCATTCAAAGAATCTCGACAAACTAGAACAACCATCTCTTGAGTTACAGCTAGTGGAGAACAGCAACTACTCCAGG**

**65 I L D R H N L H S K N L D K L E Q P S L E L Q L V E N S N Y S R**

**289 TTAAGCAAGGAAATTGCGGCAAAAAGTCATCAACTTAGGCAGATGAGAGGAGAAGAAATTCAAGGACTAAGTTTGGAAGAACTGCAACAATTGGAG**

**97 L S K E I A A K S H Q L R Q M R G E E I Q G L S L E E L Q Q L E**

**385 AAGTCCCTTGAAGCTGGCTTGGGTAGCGTAGTAGAGAAAAAGAGTGAAAAGATTAGGAAAGAGATCAACGATCTTCAAAGAAATGCGATGCAATTG**

**129 K S L E A G L G S V V E K K S E K I R K E I N D L Q R N A M Q L**

**481 GCGGAAGAGAATGAACGATTAAGAGAGCAAGTGGTGGAGAAATCTAATGGCCGGAGGCTTGTTCATGTCGATTCAGAGAACCTGATTATGGAGGAG**

**161 A E E N E R L R E Q V V E K S N G R R L V H V D S E N L I M E E**

**577 GGTCAGTCATCAGAGTCTGTCACCAATCTCTGTAAATCTAACAGCGGTCCTCAAGACCATGACAGCTCAGATACATCTCTAAAATTGGGGTGCGCT**

**193 G Q S S E S V T N L C K S N S G P Q D H D S S D T S L K L G C A**

**673 TAA**

**225 ***

**Sequence S2.** Amino acid sequence information of *EjSVP2* gene and encoded amino acid sequence

**1 ATGGCGAGGGAGAAAATTCAGATCAAGAAGATCGACAACGCGACGGCGAGGCAGGTCACCTTTTCCAAGAGGAGAAGAGGCCTTTTCAAGAAGGCC**

**1 M A R E K I Q I K K I D N A T A R Q V T F S K R R R G L F K K A**

**97 GAGGAGCTCTCCGTTCTCTGTGATGCCGATATTGCTCTTATCATATTTTCTTCCACCGGAAAGCTCTTTGAATACGCCAGCTCTAGTATGAAGGAA**

**33 E E L S V L C D A D I A L I I F S S T G K L F E Y A S S S M K E**

**193 ATTCTAGAGAGGCACAACTTGCATTCAAAGAATCTCGAGAAACTAGAACAACCATCTCTTCAGCTACAGCTAGTGGAGAACAGCAACTACACCAGG**

**65 I L E R H N L H S K N L E K L E Q P S L Q L Q L V E N S N Y T R**

**289 TTGAGCAAGGAAATTGCAGCAAAAAGTCATCAACTTAGGCAGATGAGAGGAGAAGAAATTCAAGGACTAAATTTGGAAGAACTCCAACAATTGGAG**

**97 L S K E I A A K S H Q L R Q M R G E E I Q G L N L E E L Q Q L E**

**385 AAGTCCCTTGAAACTGGCTTGGGCCGCGTAATACAGAAAAAGAGTGAACAAATCATGAAAGAGATCGGTGATCTTCAAAGAAATGGTATGCAATTG**

**129 K S L E T G L G R V I Q K K S E Q I M K E I G D L Q R N G M Q L**

**481 ATGGAAGAGAATGAACGATTAAGACAGCAAGTGGCGGAGAAATCTGATGGCCGGAGGCTTGTTCAGGTCGATTCAGAGAACATGTTTACGGAGGAG**

**161 M E E N E R L R Q Q V A E K S D G R R L V Q V D S E N M F T E E**

**577 GGTCAGTCATCAGAGTCTGTCACCAATCACTGTTACTCAAACAACGGTCCTCAAGACTACGACAGCTCAGATACATCTCTAAAATTGGGGTGCGTT**

**193 G Q S S E S V T N H C Y S N N G P Q D Y D S S D T S L K L G C V**

**673 TAA**

**225 ***

**Sequence S3.** Promoter region sequence information of *EjSVP1*

TATAGGGAAAAAAAATAGAATTTTGAGAATTAAAAAAAAAAAAAAAAGCCCAAAAAACCTTAAAAAAAAATAATTTAAATCCAACGGTAACTGGCGCCAGCTAGCCCTTTAGCCAGGAATACCAGTTTTTTTGGCCAGAGCCAGCCCTTTAGCCCTCTAGCCCTCTAGATTCCGTGGGGCCCACCAAGCCCTCTGGCCTAACCCTCGGATGGAGACGGTTTTCGGGTTATTTTTGGCCGTCTGGCCCTCTTGACCATTCGGTTGGAGATGGCCTAAAAGATACTGTTTTTATGACCCGATATTTTCCATATGTGAAAACGCAAGATTTTCAAGTCACGTTTACTTAATAGTAACGGAGAACATAAAGAATCAAACAACGTAGAAATCCAACAATTTAGGAATCGATAGCCGTATGGAATCGGACAAAAAGTCTACTTATTTTCCCTTTGAAATGGGTTTGTTCCTAGTACACGAGTTTAATTTTTCATTTTCAATAATTAGTTTTGTACAGCAAATGGGAAGATGATGAACGTTGAAGGCAAAATCTTTAGGAAGATGAATGAGACACCTTTGTACAACAAAGGCACGATGCAAGCAAAGACGAAAGTTTCATTTTACTTTATATTTTTCAGTTTTGATCTCATTTATTAACATAATAGATTACACATAGTTTATGAGTTGAGATTATTTTTTGCTAGAATTGCAAGGAAAGAGAAAACATGGTCCATGAGCCTATGAGATGAATCTAATGGTGTATACTAATCTAATTATGAAAACTTTAAATACAATTATGTGATAACATATTATACTCAAAACTTAACACATGACAAGTTTTGAGTGATTGATAGTGCTACTCAACACAGTTCAGTAACTGGTTAAGTCCCACACAAATTTTCTCATTAGGGTGATACTAGAGATATTTTTTTTATATCATATTTTATAGATTGCATGACGTGAATATTTTAAAAAGTAATCTAATCATCAATCCCTTATGATCTACAAAATATAAGTTAAATAAATAATTTATCTACCATCATTCAAAATAAAATTTAACAATCAACCGTTAAATTATATGATTTTAAAAAATATAGGTAAAAAAGAGCCTTGCTAGCATTTCTTCCTCATTATCTCAATGCTTCCTGCAGCTCAACATTTGAGTTTCACGCCATCGTTAAACCTTAAACGGCAACTAAACGGTAAAGACGTGGAAACGAGAGAAATAGTTTTGCTTCACCATAAAAAGTATCTTCTGTTTCCACACTTCCGCATGCATTCCCTCTCATTCGCTCACTCTCACACACACTCTCTCTCTCCCCACTTCTCAATGTTCTCATTGGTGTCTTTTCAGTTTTAACGCTCTGAACTCTACCCACCCATCCAAATACAGCAGCAAAACCAAAACATCTCCGCAAGATTTACTCACACTGGTAAGAACTTTGAAAGCGTCAACAAATCCCCTTTCACCTACTCAGCAAAACAATCCCTTTACCTCTTAGCGTTATCCTTTTCTTTTCTCCAGTTGGTTGGAAGCATTTCTTTCCCTCTGAGACTTGTTGGTCTTGAAACTCTGGATCTGGTTCAGAATCTAAAGATGGAGATAGAGATGGATGATGGGTCCCTTTCTCTCTTTTGATCTCTCTCTCTCTCTCTCTCTCTCTCTCTCTCTCTATCTAGAAAGCTTACACTTCATTTTTAGGGTTTGCCTTTGCCCCCCACATCCTCCAAAGATTTTTGGCTAAATTTGGCAAAATGCTGCCAGTTCTGGCACTGGACCTGTCCCTCTCCAATTAAGCCCTTCCTTCCTTTGCTTGTAATTTTTTCGCCTTTTCTGGTTAGTTTTTGGCTGATTATATTCTCTGACTCAAACATAAGCCTAAACAAAAAAGCAAAATATTCCATTCCAAAACAAGCTGTAATATTCTTCTTTTTATCTTATTTTTTCTTCTTCAGGAGAACCAGAAGAAGAAAAAGGTCGAATTAATTTAACTGAAGATTAAGAGATG

AuxRR-core

CORE (CO response element)

**Sequence S4.** Promoter region sequence information of *EjSVP2*

TTTTGGACTATGTATAGAATTATAATCACCTTTCAAAATTTATGATATTATTAACCAAAATGTGATATGAAAGTACTAATTTGTCCTTAATGACTAAAATTCTTGAACAAAGGTTTGTAACTGTAAGGGTCTCCTCAGCTGTCGCGATGATTTTAATTGTTCTGCAACCTGAATACAGTAGATATGGGTTCCTACCTAAGCAATATTGTATGAGAGGGAGACATAATTTAAAGCTAAAACTTATCCATATATACACACGTCATACGATATATATATACACACACACACACATACTTGAAGAAAATGAATAGGAATATTATCGGGGAAACTGAATATTCATACTGTTAAAGAAGAAGAGGAGGAAATGGTTCGTATTTGCGAGTTGGAGGATAGCAGGAGAAGATCGGTGTTAATACAAGAGGTTGTGATGGCAGCAGCGTTTGGGTGTCGTGGCAGCAAGAGCATTAGCCCAACTCAAAGGTGACTTAGACTTTTCACCGCCCCGCCCCACAACCACCATATCCAATTTTCCACAGCCGCTACCTACACAGCCTCCCATTCTTAGTAGATCCTCAGTCCCATTCTTCACCAGACCCTCCTCCTCCTCCATTGAAGTATAATGGGACAAAATGTTTGGTATTTCGTACGAAGAACTCTTTCTCCTCATCGGTGCCACCACTGCTCTACTCGGTACGACAGAACTACTACTTCGTTCACGATTAAGACTAGAAAGAGGATGATTTTAGTTATTCGGAGTAAATTAGTACTTTCATACCAAATTTTAGTTATAATTATAATAATCTTTAAATAGTAACTATAATTCTAAATAGAATTAAAATTTTAGTTACTTTTTCAAATTTTTCAAAACTAGCTTGAAAATTTACTTTTTACCGAATATAGTCTAAAAAAATTACGCATTTTCCATCATATCTCACTGTTTCCCTGCAGCTCAACATTTGAGTTTCACGCCATCGTTAAACCTTTTAACAGCAACAACTAAACGGTAAAACGTGGAAAAAGAGGATAGTTTTGCTTCACCATAAAAAGTATTTTCTTTTTCCACACTTCCACAGACATTCCCCCTTCTCTCTCTCTCTCTCTCCCCACTTCTCAGTGTTCTCATTGGTGGCTTATTCAGTACTAACAGCTCAGTGAACTCTATCTACCGATAGATTTT

CORE (CO response element)

CORE (CO response element)

CORE (CO response element)

TCAAGTGACCGGTACACGAAATAATACATCATGTTATATAAATGATGAGATATGTGTGTTAAAAAGTTAATAATTTAAAAATTAAAATTTCACACTACTTATATAAAAACACATAATGTATTACTCGTGTTTCTATTACAATAAAAAATTTCTCCTGTCTACCCATCCAAATACGGCACCAATACCAACACCCCTTCCCCGAGATTCAGTCAGACTGGTAAATAAATAAGTAACCACGTAAGTAAGAACTTTAAAGGCATCAACAAACAAAACCCTTTACCCCTTTTAGCTTTATGCTTTTTTTTTCTCCAATTGGTTGGGAGCATTTCTTTCCCTCTGAGCCTCGGCGGTCTTGAAACTCTGGATCTGATTAAGAATCTAAAGATGCAGATGGATGCTGGGTCTCTTTCTCTCCTTTGATATCTCTTTCTCTCTGTCTCGAAAACTTTCACTTCACTTTGACGGTTTGCCTTTGCCCCCCACATACTCCAAGGATGTTTAGCTAAATTTGGCAACATGCCGCCAGTTCTCGCACTGGATCTGTCCATCTCCAATTAAGCCTATCCTTCATTTGCTTGTAATTTCTTTGCCTTTTCTGGTCAGTTATCCTTCATTTGCTTGTAATTTCTTTGCCTTTTCTGGTCAGTTTTTGGTTGATTATATTCTCTGACTCAAACATCTAAAGCCTTAACAAGAAAGCAAAATATTCCATTCCAAAAAAAGCTATAATATTCTTATTATTTTTAATATTTTTTCTTCAGGAGAACCAGAAGAAGAAGAAGAAGAAAAGGAAGATCGAATTAATTTAATTGAAGATTAAGAGATG

CORE (CO response element)

CORE (CO response element)

CORE (CO response element)
